# Editors’ Pick: Normal aging *versus* Alzheimer’s disease – expression patterns may discern the differences

**DOI:** 10.1186/2041-2223-3-11

**Published:** 2012-05-18

**Authors:** Bruce Budowle

**Affiliations:** 1 Institute of Investigative Genetics, Department of Forensic and Investigative Genetics, University of North Texas Health Science Center at Fort Worth, 3500 Camp Bowie Blvd, Fort Worth, TX, 76107, USA

## bang

“The Times They Are a-Changin’,” one of Bob Dylan’s albums of the 1960’s, reflected the social change of the time. These same words reflect science today as it has and continues to change, providing advances that once would have been thought to be impossible or would require monumental efforts as applied to the human genome project. These tools will have a tremendous impact on society and individuals with a promise of improving the quality of life. An example of the possibilities that the wave of molecular biology advances affords is the recent publication by Podtelezhnikov *et al.*[1]. They show convincingly the value of the technology of expression arrays for studying pathways of complex diseases, such as Alzheimer’s disease (AD), that may affect many of us, either directly or indirectly, through a family member or friend.

Podtelezhnikov *et al.*[1] investigated global structure of age- and disease-dependent gene expression patterns in three brain regions from 600 individuals. The brains were obtained post mortem from individuals who died from AD, Huntington’s disease or from normal aging. They defined four metagene complexes and their variance with AD. Each metagene was a large group of genes that varied as a function of age and disease status. The first group of genes (BioAge) was related to biological age and, as expected, presented key gene expression patterns that explained the largest component of variance among the cohorts. The other three metagene groups - Alz (Alzheimer), Inflame (inflammation), and NdStress (neurodegenerative stress) - contribute less than BioAge but all together allow for detection of novel molecular biomarkers for prediction and prognosis of AD progression. The authors suggest, and rightly so, that “AD can be viewed as an aberrant aging of the brain, which retains the gene expression hallmarks of normal aging combined with additional patterns associated with pathological drivers of the disease and response of the brain tissue to disease-related processes.” Their data suggest an AD progression model of injury obviously associated with age, the primary factor, with chronic neuroinflammation, and with brain cells transitioning to a new state. While the study lacks samples from brains at early stages of AD, the data still may provide therapeutic strategies for AD lipid metabolism and inflammation. Perhaps, if such strategies could be administered at the earliest stages of AD or in individuals at high risk, the effects of AD could be delayed or, better yet, eliminated.

Yes, the times of science have changed and all for the better. We live longer today than in any time in human history and the price is that a good portion of us, almost half of us over 85, will be affected by AD. With advances in sciences, as shown in the example above, the ravaging effects of AD and many other diseases may in our lifetimes no longer be the feared afflictions that they are today.
